# The Prevalence of Depression and Acceptance Rate of Referral to Psychiatrist Among Psoriatic Patients in Saudi Arabia

**DOI:** 10.7759/cureus.63155

**Published:** 2024-06-25

**Authors:** Ahmed Baabdullah, Asail S Alghamdi, Haya Obaid, Abdulrahman A Aqeel, Ruya Abdullah, Sadeem Alqallaf, Amal Al-Sowaidi, Abdulaziz AlGhamdi

**Affiliations:** 1 Dermatology, King Abdulaziz University Hospital, Jeddah, SAU; 2 Medicine, Albaha University, Albaha, SAU; 3 Medicine and Surgery, King Abdulaziz University Hospital, Jeddah, SAU; 4 Medicine and Surgery, Jazan University, Jazan, SAU; 5 Medicine, Ibn Sina National College for Medical Studies, Jeddah, SAU; 6 Medicine, Vision College, Riyadh, SAU; 7 Medicine, King Saud University Hospital, Riyadh, SAU

**Keywords:** psychiatric, stress, acceptance rate, depression, psoriasis

## Abstract

Background

Psoriasis is a chronic inflammatory disease that primarily affects the skin and can significantly impact the quality of life. Previous studies have shown that psoriasis increases the risk of depression; however, there is a lack of information about the prevalence of depression and psoriatic patients’ acceptance of referral to a psychiatric clinic in Saudi Arabia. This study aims to estimate the prevalence of depression among psoriatic patients in Saudi Arabia, determine their referral acceptance rate to psychiatric clinics, and assess their knowledge regarding the impact of depressive symptoms on psoriasis.

Methods

This questionnaire-based cross-sectional study included patients with psoriasis who presented to dermatology clinics in Saudi Arabia. All patients were instructed to complete three sets of questionnaires encompassing sociodemographic information, the clinical characteristics of their illness, and the Beck Depression Inventory.

Results

Of 406 patients, 54.9% had symptoms of depression, and 46.3% were willing to go to a psychiatrist when symptoms of depression were present. Most of the patients (70%) thought that depression affects psoriasis.

Conclusions

More than half of the psoriatic patients in this study were depressed. The patients understood that depression can lead to a worsening of their symptoms, but the majority refused to see a psychiatrist. To remove barriers to seeing a psychiatrist, psoriasis patients should be provided with mental health assistance, taught to identify psychological stressors and effective strategies to cope with these stressors, and educated about available services, including telemedicine.

## Introduction

Psoriasis is a chronic inflammatory disease primarily affecting skin, nails, and joints. It has a bimodal onset age (16-22 years and 57-60 years) and is mediated by types 1 and 17 helper T-cells [1]. It is estimated that 2%-3% of the global population suffers from psoriasis, which can have a significant impact on a patient’s quality of life [2]. Patients with psoriasis are at an increased risk for a variety of medical conditions, including depression, anxiety, inflammatory bowel disease, malignancy, metabolic syndrome, and cardiovascular diseases. They may be predisposed to suicidal ideation and behavior (SIB). Therefore, screening patients for comorbidities is important [3,4].

The psychosocial impact of psoriasis has attracted much attention from researchers. Patients have reported feeling stigmatized and embarrassed because of their physical appearance [5]. A patient’s perception of their disease severity, regardless of the objective extent of skin involvement, correlates with depression risk [6]. The percentage of psoriasis patients with depressive symptoms ranges from 9%-55% due to differences in assessment criteria and study populations [7]. Patients with psoriasis who fail to respond to treatment have higher rates of depression and anxiety [8]. Only 40%-50% of psoriatic patients adhere to psoriasis therapies [9]. Depression in psoriasis patients may limit treatment adherence, which can result in worsening psoriasis and greater depression. The presence of psychiatric comorbidity is a major predictor of poor adherence [10]. According to a study conducted in the United States between 2009-2012, psoriasis is significantly associated with major depression, even after adjusting for sex, age, race, body mass index, physical activity, smoking history, alcohol use, history of myocardial infarction (MI), history of stroke, and history of diabetes mellitus. Moreover, no correlation was found between the severity of psoriasis and major depressive disorder. Regardless of the severity of their psoriasis, all patients who have psoriasis may be at risk for major depression [11]. 

According to a recent study, 130 of 335 psoriasis patients showed signs of depression. However, screening for depressive symptoms led to an increase in the utilization of mental health care and an improvement in psoriasis, depressive symptoms, and quality of life [12]. 

To our knowledge, no studies focusing on the prevalence of depression in psoriatic patients and the referral acceptance rate of psoriatic patients to psychiatry clinics in Saudi Arabia are available. Therefore, we aimed to conduct a cross-sectional study to answer these questions.

## Materials and methods

This study was cross-sectional in nature. The primary objective was to estimate the prevalence of depression among psoriasis patients in Saudi Arabia. The secondary objective was to determine the referral acceptance rate of psoriatic patients to psychiatric clinics and to assess the knowledge of psoriatic patients regarding the impact of depressive symptoms on psoriasis. Ethical approval was obtained from the research ethics committee of King Abdulaziz University (reference number 474-22).

The study population included psoriatic patients across Saudi Arabia. Using the Cochran formula, a minimum sample size of 377 psoriatic patients with a confidence level of 95% and a margin error of 5% was calculated.

Both Saudi and non-Saudi psoriatic patients attending dermatology clinics in Saudi Arabia between September 2022 and November 2022 who had not previously been diagnosed with depression or other psychiatric issues were included in the study. Patients with other illnesses or conditions that contribute to the risk of developing depression, pregnant women, and Saudi psoriatic patients living outside of Saudi Arabia were excluded. The patients who met the inclusion criteria and agreed to participate completed an anonymous questionnaire involving demographic data, screening-related questions, and aggravating factors.

To determine the prevalence of depression, the Beck Depression Inventory was used. This is a 21-item questionnaire; each item was scored from 0-3 based on symptom severity (absent, mild, moderate, or severe), with a total score ranging from 0-63. A score of less than 26 was considered normal, 26-28 indicated mild depression, 39-55 indicated moderate depression, and 56-63 indicated severe depression [13].

Two different individuals performed the data entry. The data were cleaned and verified and then directly transferred to a statistical database. The data were analyzed statistically using the SPSS (IBM Corp. Released 2019. IBM SPSS Statistics for Windows, Version 26.0. Armonk, NY: IBM Corp). To test the relationship between variables, the qualitative data was expressed as numbers and percentages, and the Chi-squared test (χ2) was used. The quantitative data was expressed as mean and standard deviation (Mean ± SD); a p-value of less than 0.05 was considered statistically significant.

## Results

A total of 406 psoriasis patients who presented to dermatology clinics in Saudi Arabia were included in this study. 

The demographic characteristics are listed in Table 1. About 44.6% were 26-65 years old, 64% were female, 90.1% were Saudi, 74.1% had a university-level education, 32.8% were students, and 53.9% were single. Almost one-third (36%) had comorbidities; other skin diseases were the most common comorbidity (23.2%). For patients (n = 146) with comorbidities, 49.4% reported that they had these diseases before the onset of psoriasis.

**Table 1:** Patient demographic data and comorbidities distribution GIT: gastrointestinal tract; DM: diabetes mellitus; HTN: hypertension

Variable	No. (%)
Age (years)	
18–25	223 (54.9)
26–65	181 (44.6)
<66	2 (0.5)
Gender	
Female	260 (64)
Male	146 (36)
Nationality	
Saudi	366 (90.1)
Not Saudi	40 (9.9)
Educational level	
Illiterate	2 (0.5)
Primary and middle	11 (2.7)
Secondary	92 (22.7)
University	301 (74.1)
Employment	
Student	133 (32.8)
Unemployed	99 (24.4)
Government employee	96 (23.6)
Private sector employee	78 (19.2)
Marital status	
Widow	3 (0.7)
Single	219 (53.9)
Married	167 (41.1)
Divorced	17 (4.2)
Comorbidities	
Yes	146 (36)
No	260 (64)
If yes, specify:	
Skin disease	94 (23.2)
GIT disease	30 (7.4)
Chest disease	14 (3.4)
Reproductive problems	4 (1)
Immunological disease	10 (2.5)
DM	5 (1.2)
Cardiac disease	9 (2.2)
Anemia	1 (0.2)
Hypothyroidism	3 (0.7)
HTN	4 (1)
Genitourinary disorder	2 (0.5)
Musculoskeletal disorder	2 (0.5)
When did these diseases (chronic diseases) begin: (No. 146)	
After the onset of psoriasis	31 (21.2)
Before the onset of psoriasis	72 (49.4)
There are no comorbidities	19 (13)
With the appearance of psoriasis	24 (16.4)

The questions related to the duration of and factors that aggravate psoriasis are listed in Table 2. About 83.5% of the participants had psoriasis for more than a year, and 81.8% reported the presence of factors that increase psoriasis symptoms. The two most common factors were psychological stress-related factors (64.1%) and winter (43%). With regard to severity, 35.2% of the participants reported mild psoriasis, 40.1% reported moderate psoriasis, and 50.7% were undergoing treatment.

**Table 2:** Patient distribution of duration of and factors aggravating psoriasis

Variable	No. (%)
Psoriasis duration	
More than a year	339 (83.5)
Less than a year	67 (16.5)
Are there factors that increase your psoriasis symptoms?	
Yes	332 (81.8)
No	74 (18.2)
If yes, specify: (No. 332)	
Psychological stress	213 (64.1)
Winter	143 (43)
Summer or exposure to sunlight	125 (37.6)
Smoking or alcohol	41 (12.3)
Inflammation, such as inflammation of the tonsils or throat	32 (9.6)
How severe is your psoriasis?	
Mild	143 (35.2)
Moderate	163 (40.1)
Severe	65 (16)
Don't know	35 (8.6)
Are you currently using a treatment for psoriasis?	
No	200 (49.3)
Yes	206 (50.7)

When asked about follow-up care, 44.1% of the patients reported the ease and accessibility of treatment as moderate, and 40.1% reported the ease of scheduling an appointment with a specialist as moderate. Around 49% (49.8%) reported having a good relationship with their specialist. Only 46.3% were willing to go to a psychiatrist when experiencing symptoms of depression. For those who were not willing to see a psychiatrist, the most common barrier was not feeling the need. Most of the patients (70%) thought that depression affects psoriasis, and 62.8% thought that if their depressive symptoms were treated, their psoriasis would improve (Table 3).

**Table 3:** Patient distribution for follow-up care

Variable	No. (%)
How would you describe the ease and accessibility of treatment?	
Easy	156 (38.4)
Moderate	179 (44.1)
Difficult	71 (17.5)
How would you describe the ease of scheduling an appointment with a specialist?	
Easy	144 (35.5)
Moderate	163 (40.1)
Difficult	99 (24.4)
How is your relationship with your specialist?	
Excellent	159 (39.2)
Good	202 (49.8)
Bad	45 (11.1)
Do you go to a psychiatrist when you feel symptoms of depression?	
Yes	188 (46.3)
No	218 (53.7)
If you do not want to go to a psychiatrist, why not? (218)	
Cost of treatment	50 (22.9)
Social stigma	26 (11.9)
There is no need to go to a psychiatrist	105 (48.1)
The clinic is far from home	34 (15.5)
Other	44 (20.1)
Do you think depression affects psoriasis?	
Yes	284 (70)
No	122 (30)
Do you think that your psoriasis will improve if your depressive symptoms are treated?	
Yes	255 (62.8)
No	151 (37.2)

**Figure 1:**
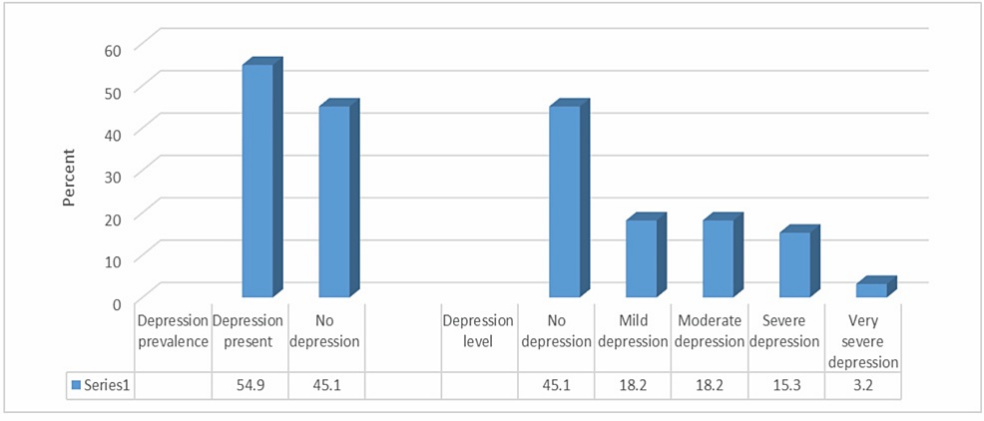
Percentage distribution of patients according to depression prevalence and severity

Using the Beck Depression Inventory, the mean depression score was 12.86 ± 10.83. Figure 1 illustrates that the prevalence of depression was 54.9%; 36.4% of the participants had either mild or moderate depression, while 15.3% and 3.2% had depression that was severe or very severe, respectively.

The prevalence of depression was significantly higher among psoriatic patients with a university education and those who were students, single, or had cardiac diseases (p ≤ 0.05; Table 4). The prevalence of depression was significantly higher among psoriasis patients who reported the presence of factors that increase psoriasis symptoms, especially those who reported experiencing psychological stress. Depression was reported to be significantly higher among patients with mild psoriasis (p ≤ 0.05; Table 5) as well as those who experienced moderate ease and accessibility to treatment, moderate ease in terms of the possibility of scheduling an appointment with a specialist, and had a good relationship with their specialist (p ≤ 0.05; Table 6).

**Table 4:** Relationship between depression prevalence and patients' demographics and comorbidities

Variable	Depression		χ2	p-value
	Absent No. (%)	Present No. (%)		
Age (years)				
18–25	97 (53)	126 (56.5)	0.5	0.777
26–65	85 (46.4)	96 (43)		
66–79	1 (0.5)	1 (0.4)		
Gender				
Female	111 (60.7)	149 (66.8)	1.65	0.198
Male	72 (39.3)	74 (33.2)		
Nationality				
Saudi	170 (92.9)	196 (87.9)	2.83	0.092
Not Saudi	13 (7.1)	27 (12.1)		
Educational level				
Illiterate	1 (0.5)	1 (0.4)	9.04	0.029
Primary and middle	1 (0.5)	10 (4.5)		
Secondary	35 (19.1)	57 (25.6)		
University	146 (79.8)	155 (69.5)		
Employment				
Student	63 (34.4)	70 (31.4)	11.69	0.008
Unemployed	32 (17.5)	67 (30)		
Government employee	54 (29.5)	42 (18.8)		
Private sector employee	34 (18.6)	44 (19.7)		
Marital status				
Widow	0 (0.0)	3 (1.3)	20.5	<0.001
Single	85 (46.4)	134 (60.1)		
Married	95 (51.9)	72 (32.3)		
Divorced	3 (1.6)	14 (6.3)		
Comorbidities				
Yes	57 (31.1)	89 (39.9)	3.35	0.067
No	126 (68.9)	134 (60.1)		
If yes, specify:				
Skin	36 (19.7)	58 (26)	2.26	0.132
GIT	14 (7.7)	16 (7.2)	0.03	0.855
Chest	4 (2.2)	10 (4.5)	1.59	0.207
Reproductive	1 (0.5)	3 (1.3)	0.65	0.417
Immunological	2 (1.1)	8 (3.6)	2.6	0.107
DM	3 (1.6)	2 (0.9)	0.45	0.5
Cardiac	1 (0.5)	8 (3.6)	4.28	0.038
Anemia	1 (0.5)	0 (0.0)	1.22	0.269
Hypothyroidism	0 (0.0)	3 (1.3)	2.48	0.115
HTN	3 (1.6)	1 (0.4)	1.46	0.227
Genitourinary	1 (0.5)	1 (0.4)	0.02	0.888
Musculoskeletal	1 (0.5)	1 (0.4)	0.02	0.888
When did these diseases begin? (No.146)				
After the onset of psoriasis	16 (8.7)	15 (6.7)	7.28	0.122
Before the onset of psoriasis	23 (12.6)	49 (22)		
There are no comorbidities	7 (3.8)	12 (5.4)		
With the appearance of psoriasis	11 (6)	13 (5.8)		

**Table 5:** Relationship between depression prevalence and psoriasis duration, aggravating factors, severity, treatment use No.: number

Variable	Depression		χ2	p-value
	Absent No. (%)	Present No. (%)		
Psoriasis duration				
More than a year	148 (80.9)	191 (85.7)	1.66	0.197
Less than a year	35 (19.1)	32 (14.3)		
Are there factors that increase your psoriasis symptoms?				
Yes	139 (76)	193 (86.5)	7.56	0.006
No	44 (24)	30 (13.5)		
If yes, specify: (No. 332)				
Psychological stress	86 (47)	127 (57)	3.99	0.046
Winter	56 (30.6)	87 (39)	3.11	0.077
Summer or exposure to sunlight	58 (31.7)	67 (30)	0.12	0.72
Smoking or alcohol	21 (11.5)	20 (9)	0.69	0.404
Inflammation, such as inflammation of the tonsils or throat	14 (7.7)	18 (8.1)	0.02	0.875
How severe is your psoriasis?				
Mild	76 (41.5)	67 (30)	12.23	0.007
Severe	20 (10.9)	45 (20.2)		
Don’t know	20 (10.9)	15 (6.7)		
Moderate	67 (36.6)	96 (43)		
Are you currently using a treatment for psoriasis?				
No	91 (49.7)	109 (48.9)	0.02	0.865
Yes	92 (50.3)	114 (51.1)		

**Table 6:** Relationship between prevalence of depression, referral acceptance rate of psoriatic patients to psychiatric clinics, and knowledge of the impact of depressive symptoms on the disease No.: number

Variable	Depression		χ2	p-value
	Absent No. (%)	Present No. (%)		
How would you describe the ease and accessibility of treatments?				
Easy	88 (48.1)	68 (30.5)	13.22	0.001
Difficult	26 (14.2)	45 (20.2)		
Moderate	69 (37.7)	110 (49.3)		
How would you describe the ease of scheduling an appointment with a specialist?				
Easy	80 (43.7)	64 (28.7)	10.14	0.006
Difficult	37 (20.2)	62 (27.8)		
Moderate	66 (36.1)	97 (43.5)		
How is your relationship with your specialist?				
Good	83 (45.4)	119 (53.4)	12.02	0.002
Bad	13 (7.1)	32 (14.3)		
Excellent	87 (47.5)	72 (32.3)		
If you do not want to go to a psychiatrist, why not? (218)				
Cost of treatment and sessions	21 (25.6)	29 (21.8)	0.41	0.521
Social stigma	10 (11.8)	16 (11.8)	0.001	0.999
There is no need to go to a psychiatrist	39 (45.9)	66 (48.4)	0.14	0.701
The clinic is far from home	18 (21.2)	16 (11.8)	3.55	0.059
Other	14 (16.5)	30 (22.1)	1.02	0.311
Do you think that your psoriasis will improve if your depressive symptoms are treated?				
No	77 (42.1	74 (33.2)	3.4	0.065
Yes	106 (57.9)	149 (66.8)		

**Figure 2:**
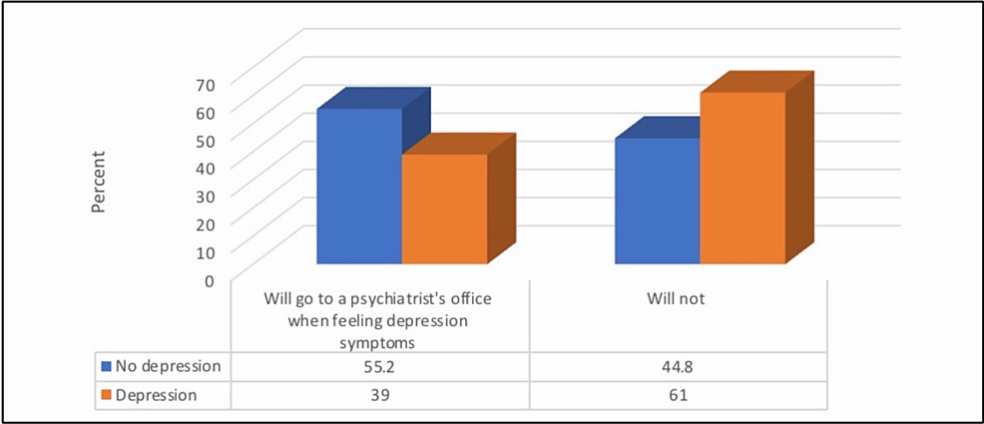
Relationship between depression prevalence and the willingness to go to a psychiatrist's office when feeling depression symptoms

**Figure 3:**
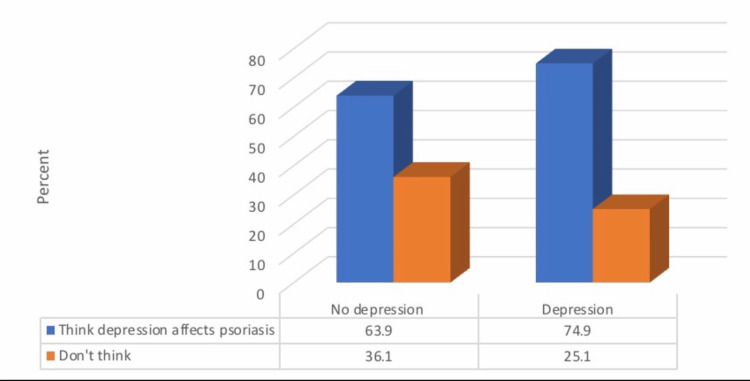
Relationship between depression prevalence and if the patients think depression affects psoriasis

There was a significantly higher prevalence of depression among patients who would not go to a psychiatrist’s office when experiencing symptoms of depression and those who thought that depression affects psoriasis (p ≤ 0.05; Figures 2, 3).

## Discussion

In this study, 54.9% of the participants had symptoms of depression, indicating that the prevalence of depression among psoriasis patients in Saudi Arabia is quite high and warrants attention. The majority (81.8%) of the participants reported the presence of factors that can increase psoriasis symptoms. The most common was psychological stress (64.1%), indicating that psoriatic patients are aware of the effect of stress on their disease.

Among the participants, 44.1% reported the ease and accessibility of treatment as moderate, and 40.1% reported a moderate level of ease related to scheduling an appointment with a specialist. However, 17.5% reported difficulties with treatment accessibility, and 24.4% reported difficulties scheduling an appointment with a specialist. These findings should not be ignored due to the importance of treatment in preventing disease progression.

The majority (53.7%) of the participants reported that they would not go to a psychiatrist if they felt symptoms of depression. The most common barrier (48.1%) was not feeling the need to go to a psychiatrist. Cost of treatment was the second most common barrier (22.3%); however, most participants were Saudi, which means they are eligible for free services provided by the Ministry of Health. This finding may indicate a lack of knowledge about the available services. The third most common barrier (15.5%) was the distance to the clinic from patients’ homes. However, the Ministry of Health provides telemedicine options for many services, further indicating that patients may not be fully aware of the available services. The least common cause was shyness (11.9%), which may indicate that the stigma associated with psychological diseases is starting to decrease.

A significant relationship (p = 0.05) was found between patients who refused to go to a psychiatrist when experiencing depressive symptoms and those who believed that depression affects psoriasis, suggesting that there are reasons for not seeking treatment for depression other than a lack of knowledge about the relationship between the two conditions.

In a 2012 study of psoriatic patients that examined the degree of depression and anxiety disorders using the Beck Depression Inventory and the Spielberger State-Trait Anxiety Scale I-II, psoriatic patients reported significantly higher degrees of depression and anxiety than the controls [14]. A 2017 study that also used the Beck Depression Inventory concluded that patients with psoriasis, irrespective of severity and related complications, are at an increased risk of depression [15]. These studies are consistent with our findings, as the majority of psoriatic patients in our study experienced some level of depression. Taken together, these studies indicate that this disease requires multidisciplinary approaches to cover both the cutaneous and psychological aspects.

This study highlights the prevalence of depression, the low acceptance rate regarding visiting a psychiatrist, and knowledge levels regarding the effects of depression on the disease among psoriasis patients. It also demonstrates the need to raise awareness of the free services provided by the Ministry of Health and the availability of telemedicine, which could be especially helpful for patients who live in rural areas that are far from psychiatric clinics.

Future studies should explore psoriasis patients’ knowledge levels about telemedicine and its uses and dermatologists’ and family physicians’ awareness of depression screening practices when treating psoriatic patients.

In spite of a high response rate, the limitations of this study include recall and response bias.

## Conclusions

In this study, more than half of the participants suffered from depression. Although the psoriatic patients were aware that depression can negatively affect psoriatic symptoms, the majority refused to go to a psychiatrist when they experienced depression. It is important to remove the obstacles preventing patients from visiting a psychiatrist when needed. Psoriasis patients should be provided with mental health support, helped to identify psychological stressors and ways to cope with them, and educated about the available services, including the existence of telemedicine.
